# Bibliographical Notices

**Published:** 1849-02

**Authors:** 


### BIBLIOGRAPHICAL NOTICES.

*An Illustrated System of Human Anatomy, special, general and microscopic.* By SAMUEL GEORGE MORTON, M. D. Pennsylvania and Edinburgh. Member of the Medical Societies of Philadelphia, New York, Boston, Edinburgh, and Stockholm; author of "Crania Americana," "Crania Ægyptiaca," &c. With three hundred and ninety-one engravings on wood. "Oculis subjecta fidelibus." Royal 8vo. pp. 642. Philadelphia, 1849.

How essentially different are the treatises on anatomy of the present day from what they were even thirty years ago! Illustrated works were rare, and when they did appear "*in gurgite vasto*," their price effectually prevented them from being available to the student. Now, the best works on the subject are so interlarded with illustrations, that there is scarcely a topic, even of minute structure, that has not the xylographic figure to address the eye, and thus to render comprehensible what no unaided powers of language could possibly make entirely so. This is a vast improvement over ancient methods; and the advantage to the anatomical student is almost inappreciable, especially in regard to subjects of microscopic investigation. In the parts as exhibited on dissection he has always a mode of verifying the statements of authors; but not always so where the microscope is concerned. Carefully executed sketches of the appearances presented in the field of that instrument are, consequently, all important; and, thanks to the zeal of observers! these now abound; and are everywhere attainable.

It is instructive to cast a glance over anatomy as taught before the present century; and to contrast it with the existing system. Then, the labour of the student was almost wholly restricted to descriptive anatomy; and if he could name the numerous processes of the sphenoid bone, and the arrangement of other parts of the frame of an equal degree of minuteness, he was regarded as an accomplished anatomist. General anatomy was hardly known, or in its infancy only; its developement, which had lain for some time dormant, acquired fresh vigour under the genius and fostering industry of Bichat; and of late years its whole face has been modified by the investigations of the microscopist. Valuable, however, as have been the additions made by recent observers, great caution is needed in assuming the results as in all cases established; and in placing a proper estimate on such results where they even seem to be unquestionable. The promulgation of the cell-doctrine was regarded by many—to our own knowledge—as leaving but little more for the investigations of the biologist. Every thing was believed to be comprised in the idea of cell-life—even the essence of life itself. Sober-minded individuals, however, considered—and yet consider—that we are no nearer the solution of the great problem of life than we were prior to the researches of Schwann; and almost all physiologists—reflecting and observing physiologists, that is—are disposed to regard his generalizations, as to the universality of cell-agency in the formation of the tissues, premature. Simple membrane, in their view, neither exhibits, nor does it need, nucleated cells.

The doctrine of cell-agency certainly explains many living acts, which otherwise would be sufficiently unaccountable; but it is invoked to solve difficulties for which it is but little adapted; and, as with the terms *vital*, *organic*, &c, when applied to phenomena that cannot be likened to any thing physical, it is occasionally used somewhat cabalistically; and apparently to cut a Gordian knot, when the utterer is unable to untie it satisfactorily.

Nor are we sure that the enthusiasm, occasioned by the wonderful display of microscopic observations in modern times, has not been the parent of evil. Phenomena are doubtless essential to the formation of every science; but the simple observation of phenomena is not science; nor is the observer of phenomena necessarily scientific: he is but the pioneer of science. The collector of a museum of natural objects is a most useful minister; but he may be a mere virtuoso,—as the collector of a museum of pathological specimens may be anything but a pathologist. So is it with microscopic and other observers: they are most valuable in their vocation; but they may be utterly unfit even to classify what they observe, and still more unfit to detect laws of phenomena that may tend to enlarge the boundaries of science. "They may,"—to employ language ascribed to the illustrious Franklin, as applicable to the people of a certain portion of this continent—" be able to raise onions, but they cannot string them."

To observe—to proceed one step farther, and to classify—are necessary to the philosopher who attempts to deduce laws or to constitute a science; but where there are no laws of phenomena there can be no science; no matter what may be the number of phenomena observed, or the arrangement given to them. This we hold to be a self-evident proposition.

The great characteristic of the medical mind, in modern times, has been to observe; and it must always happen, that observers will be numerous in proportion to philosophers. It is easier, for example, to mark the state of the thermometer, barometer, hygrometer and anemometer daily, and several times a day, than it is to reflect on the useful results to which such observations may lead. It is much less difficult, in other words, to *see* or to *hear* than to *think*. Hence the multitude of meteorological registers kept for years by persons who, to use the language of a literary—if not scientific—leviathan, have died fully convinced, from their observations, that the wind is changeable! They are mere automata, and their tables, on which they have spent so much irreclaimable time, are, in reality, not calculated to elevate them above the class of "idlers," and could have been as satisfactorily accomplished by hired and almost uneducated domestics as by themselves. The purchase of the necessary instruments does not constitute them men of science; and still less can such observations, made by themselves, or under their direction by proxy, be esteemed scientific; notwithstanding they may form elements from which the really scientific may be enabled to deduce laws of science. Hence it is, that the old methods of keeping meteorological records, excepting as a simple registry of facts or phenomena applicable to localities, has been generally abandoned by philosophers, and a system of simultaneous observation has been substituted, which *may* enable laws of atmospheric phenomena to be established, and thus give to the *indigesta moles* a scientific form and feature.

We think that we are able to perceive in the tentative or experimental methods of the day, in all sciences, a signal improvement. Instead of vague and disconnected observation, unsuggested by any rational hypothesis, the philosopher now—as he ought always to have done—sets out with a preconceived idea, the result of profound thought and careful examination of every fact or phenomenon, that can have any—the most remote—bearing on the subject of his inquiry: he observes and compares the recorded observations of others; and if, after a sedulous examination into every possible source of fallacy, he finds that the observations confirm and establish his hypothesis, he correctly infers, that he is justified in regarding that, which was at first hazarded as an hypothesis, to be a law of phenomena, and a solid addition to science. If, on the other hand, the result of reiterated observation of phenomena does not support—and *à fortiori* if it negatives—his preconceived hypothesis, he unhesitatingly rejects it, and substitutes another, which has to be subjected to the same scrutiny; and thus he proceeds until at length he succeeds in framing and establishing one that receives unquestionable support from observation

In anatomical investigations of recent date, attention has been directed mainly to the results of observation by the microscope and organic chemistry. The beautiful and accurate achromatic instruments, from the workshops of the English mechanicians more especially, have enabled the histologist to unravel, with something like certainty, the precise arrangement of formations—healthy and morbid—which had not been previously appreciated; whilst the chemical analyst has exhibited the intimate composition of the tissues of organized bodies, and tended to simplify much that was previously enveloped in complexity and obscurity. Still, the results of microscopic inquiry have been—in too many cases—more striking and showy than ancillary to the great object of the scientific biologist—to fathom the laws of life. As in the case of colossal representations by the solar microscope, for the first time unveiled to the gaze of uninitiated spectators, the hands are elevated in astonishment; and the mind is disturbed and harassed at the aspect of myriads of living creatures swallowed by them unconsciously in their daily food, yet no mental impression remains calculated to materially enlarge the intellectual sphere;—so, with the mass of microscopic observers, the instrument has been procured and employed more as an article of amusement, or of virtu; and many a possessor of a most expensive and admirably fashioned instrument has self-complacently supposed, that his ability to purchase and possess the means by which valuable observations *may* be made is sufficient to rank him as an observer, and therefore a scientific physicist. The fashion of the day is for microscopic observation; but as the instrument is very expensive, when of the best construction, it is beyond the means of many; hence, every anatomical inquirer cannot be an observing histologist; and hence again, some who would, or might, be the very best observers, cannot have an opportunity of exhibiting their powers. Their condition is the reverse of that of the starved apothecary in "Romeo and Juliet": "Their will but not their poverty consents." Such being the case, need we be surprised at the number of imperfect observations that are foisted upon us! The desire of originality impels to the publication of crude and ill-digested results; for it is proverbial, that they who are the most superficial among observers always form their opinions most speedily:—"Qui ad pauca respiciunt, de facili judicant."

Whilst we would, by no means, damp the ardour of microscopists, we may be permitted to express our decided conviction, that too much has been, and is, expected from their labours. Every lustre brings up some new notion, which at once becomes a hobby, and is spurred and belaboured, until it either unhorses the rider, or is cast aside in disgust; and, although calculated—if properly managed, in the first instance—to arrive at some desirable goal, breaks down and falls into total and often unmerited neglect. To speak of recent times:—at one period, Broussaism was not merely the fashion, but the rage; at another, hematology; at another, the formation of cabinets of pathological specimens, arranged as in museums of natural history, to be gazed at—too rarely to be studied. And what is the history of all these 'rages'? Very much like that of *some*—by no means *all*—of those instanced by a cynical but gifted British poet:

> "The cowpox, tractors, galvanism and gas
> In turns appear to make the vulgar stare:
> Till the swol'n bubble bursts and all is air."

Yet, who can doubt, that if sober zeal had occupied the place of transient and ill-directed enthusiasm, each of these 'rages' might have left more signs, when supplanted by a rational and enduring system of observation and reflection.

We think, that the respectable author of the work before us is amongst those who expect too much from microscopic anatomy. He says—and we would not gainsay this—that "it presents a new field for discovery, replete with surprising and instructive revelations;"—but we are not so sure, that we can accord with him, that "it has already banished a legion of venerable conjectures, and substituted simple truth for vague hypothesis; and, by unveiling the very germs of organized and organizable [?] matter, has taught us the origin and development of the tissues, together with a portion, at least, of their hitherto doubtful functions."

A like exaggerated opinion has, it seems to us, been entertained in regard to the information capable of being afforded to the biologist by organic chemistry. Undoubtedly it has enlarged, and will continue to enlarge our sphere of knowledge as to the intimate composition of *dead* organized matter; but, it must always be borne in mind, when we apply the knowledge, thus afforded, to *living* phenomena, that the same problem does not exist in the two cases.

The world—the οἱ πολλοι—the learned and the unlearned—were astounded at the categorical promulgations of Liebig, who, from the crucible, as it were, oracularly expounded the laws of life, in a manner that seemed scarcely to admit of contestation; yet, although a very short time has elapsed since he dazzled us with his meridian splendour, obscuring—if we may be permitted the expression—by his greater brilliancy, the lesser lights of the chemical firmament, causing them "to pale their ineffectual fires"—how few of his results can be regarded by the biologist as undisputed landmarks in the realm of science.

It is then against an overestimate of the importance of these and other modes of scientific investigation of the phenomena of biotics that we would caution the younger members of the profession more especially; for such overestimates must inevitably lead to disappointment; whilst we would encourage them to employ *every* agency at their command, and especially the two which we have instanced—microscopy and organic chemistry—with a zeal that never flags; and a fidelity that does not admit of cavil.

But we must proceed to a notice of the imposing and well executed volume, which has suggested the preceding observations. The title is sufficiently explanatory of its scope; and its objects are clearly set forth in the preface. "In preparing this work for the press," says the author, "I have been influenced by the usual incentives to publication, one of which, and not the least, is the desire to be enrolled among the expositors of a science that has occupied many of the best years of my life. I need not be reminded, that my enterprise must necessarily be devoid of that originality which gives charm to authorship; for a writer on special anatomy in our day must, for the most part, be content to clothe familiar facts in new language. Nor is even this object always attainable in a path that has been trodden for centuries by ambitious and scrutinizing minds, who, like voyagers in search of undiscovered lands, have left comparatively little to reward the zeal of their successors:"—and in speaking of microscopic anatomy he adds—"In this department, however, I have not entered the lists as a discoverer, but as a learner; for although I commenced with the intention of furnishing a series of original drawings, I soon found that it required a more practised eye and hand than mine, to do justice to these delicate manipulations, and that I must be content with the humbler task of occasionally verifying the observations of others."

The work—from its dimensions—could obviously only admit of an epitome of histological observations, and therefore they who expect detail may be disappointed. Great efforts have manifestly been made to reconcile conflicting statements; and although, at times, some degree of obscurity may still remain, we are well aware, that much of this depends upon the very nature of the subject, and upon the varying testimony of observers—who, notwithstanding, are "all honorable men." As an instance of want of perspicuity, we would specify the descriptions of nucleoli on the first page, under the head of "Blastema," and on the subsequent page;—the omission of any reference to nuclei in the first page being calculated to lead to confusion in the minds of those for whom the work is particularly intended, and to whom it is dedicated—"students of medicine."

It is impossible for us to enter into any analysis of it. It considers, in succession, Histogeny, Osteology, Odontology, Arthrology, Dermology, Myology, Aponeurology, Splanchnology, Angeiology, and Neurology.

The descriptions of the different parts of the human fabric are accompanied by necessarily brief expositions of their physiological manifestations and relations.

"The present state of anatomical science," says the author, "forbids its entire separation from physiology, inasmuch as the results of microscopic discovery have more than ever blended the functions of parts with their structure [?] A glance, however, at this work, will show, that physiology has been therein considered in a merely elementary manner, and as far as possible with regard to facts alone, without reference to doubtful propositions; for my aim has rather been to invite, on the part of the student, increased attention to this pleasing and all-important branch of professional learning, as more fully elucidated in the several works referred to in the following pages." p. viii.

The work is beautifully got up, and most of the illustrations, which are numerous, are well selected to elucidate the text. The author was for years engaged as an anatomical teacher, and is, therefore, an advanced student—for we regard the most accomplished teacher of science to be such, and nothing more :—" qui docet discit." He is, however, admitted—here, and everywhere—in this country and in all countries—to be a skilful anthropological observer—the justly distinguished author of two works—" Crania Americana," and " Crania Ægyptiaca "—which are monuments of zeal and industry, skilfully and accurately directed; and we are of opinion, that in the present production he has attained the great objects with which he professes to have undertaken it.

" I have only to add," he says, " that it has been my constant aim, to convey clear and concise impressions of the structure and functions of the various tissues, and I shall feel sincere gratification, if my work may happily tend to facilitate the studies of those to whom it is dedicated." p. ix.

We have noticed a few inaccuracies of expression, and in proper names. For example :—" Mr. Hassell," he says, " observes, that the orifices of these tubular processes " [of the epidermis] " are immense, [?] not less, indeed, than 3000 to the square inch "! (p. 149.) " *Gastro-enteritic* " for gastro-enteric, (p. 311.) At page 317, the reference to fig. 219 is evidently a dislocation. " *Demarcay* " is written more than once for Demarçay. From the note at page 379, it might be supposed, that hanging, strangulation and smothering, are not causes of asphyxia :—" In asphyxia, there is a total suspension of the respiratory function, either from the presence of water as in drowning, or from breathing of carbonic acid gas, or other deleterious vapours." The description of the action of the heart, at page 394, requires—to employ an expressive nautical term—" overhauling." We cannot accord, that "the auricular and ventricular sounds [?] follow each other with great regularity and without any appreciable pause or interval; but the time occupied by the act of contraction [Qr. of auricles or ventricles ?] is estimated at double that of dilatation;"—nor that "the cineritious neurine of the spinal cord is now regarded as a connected chain of ganglia, whose function is to regulate the sensations and voluntary motions of the limbs and body"! (p. 490.) Neither do we exactly see how "the nerves, which the sympathetic supplies to the viscera, appear to be, though in variable degrees, the instruments of both sensation [?] and motion, independently of the will." (p. 536.) The credit of the doctrine of reflex nervous actions is given to Sir Charles Bell instead of to Dr. Marshall Hall. (p. 494.) But these, and other inadvertencies of fact, thought or expression, can be readily modified in a new edition, which, we sincerely hope, may be soon called for.

---

*Chemical and Pharmaceutic Manipulations: A Manual of the Mechanical and Chemico-Mechanical operations of the Laboratory. Containing a complete description of the most improved Apparatus, with instructions as to their application and management, both in the Manufacturing processes, and in the more exact details of Analysis and Research. For the use of Chemists, Druggists, Teachers and Students.* By Campbell Morfit, Practical and Analytic Chemist, author of "Applied Chemistry," etc. Assisted by Alexander Mucklé, Chemical Assistant in Professor Booth's Laboratory. With four hundred and twenty-three illustrations. Philadelphia: Lindsay & Blakiston, 1849.

We have often heard apothecaries lament that they could not have access to any work which would give a comprehensive account of the more difficult processes of their profession, and which would at the same time, furnish a view of the manipulations, by means of which the analytical and practical branches of chemistry proper are pursued in the laboratory. Physicians, who so often are—or, at least, should be—engaged in pharmaceutical and chemical pursuits, and in fact, all persons whom taste or business lead to take an interest in chemistry, must have sensibly felt this want. No one of the ordinary works accessible to the student, imparts the kind of information desired upon these subjects, and the only one of high character which does, and of which we have any knowledge—that of Faraday—however excellent, is not of general utility, the writer having confined his attention to those operations which concern alone the investigator and analyst.

The book of which we have given the title at the head of our notice, seems to us to supply these deficiencies completely. It is admirably adapted to the wants of the practical man; and the author, while avoiding the extreme of giving his production a strictly scientific character, has contrived to compress into four hundred and seventy pages an amount of instruction which must be of the greatest assistance to the student of experimental philosophy and analysis.

The modes of expediting and making more easy pharmaceutical processes and scientific researches, have been so abundantly increased of late, that one who is familiar alone with the apparatus in use for all such purposes ten years ago, would, at the present day, find himself quite at a loss among the multiplicity of new inventions to be found in a well furnished laboratory. Information concerning the manufacture and use of all such improved instruments, and much other matter of interest and novelty, are to be met with in Mr. Morfit's book, and are given in a way calculated to meet the wants of all. His descriptions are clear and graphic, and while adapted to the comprehension of most readers, are sufficiently minute and exact to make the book serve as a manual for the professional chemist.

We have been particularly pleased with the arrangement adopted by the author in treating of the processes of the chemical workshop. He first describes in full a model laboratory, with the nature and position of its various appurtenances, not meaning to imply that the particular conveniences mentioned by him are essential in all cases, but only with the view of giving such instruction as will enable the beginner to select for himself the elements of an effective chemical armament. He then commences with the treatment of substances designed for experiment or manufacture, and passes on through the various operations which are likely to be employed, in their order of sequence, introducing under each heading the engravings and description of the appropriate apparatus. In this way, the chapter on "The Division of Substances" is succeeded by a full consideration of the balance, weights, weighing, specific gravity, measuring, the sources, management and estimation of heat, baths, freezing mixtures, fusion, ignition, cupellation, sublimation, distillation, lutes, solution, evaporation, crystallization, desiccation, precipitation, &c. &c. This arrangement is well calculated to lead the student on *seriatim* through the various processes required to be performed by him, and also to enable the manufacturer to select with ease those parts of operations which belong to his own especial province. A list of test bottle series, and valuable tables of thermometrical equivalents, of freezing mixtures, and of the solubilities of various salts, are included in this part of the volume.

Besides the subjects mentioned above, there is an account of analysis by the polarisation of light, furnished by Professor Reynolds, of this city. Electricity and galvanism, occupy a considerable space, and treatises on the blow-pipe, glass-blowing, the construction of formulæ, and the management of corks, follow the chapter on the former subjects.

A list of the manufactures of, and dealers in the various apparatus mentioned by the author, and a copious index, conclude the work.

The author seems to have departed somewhat from his proper sphere in the chapters upon the various kinds of electricity, and in some other parts of the book, by a reference to theory which is a little at variance with the title of "Manipulations" upon his first page. This may, perhaps, be justified in the former instances, by the fact that a knowledge of the origin and action of the electric influence is essential to the right use of the apparatus intended for its exhibition. We know that very many who are in the habit of experimenting with electric machines and galvanic batteries, are totally ignorant of the nature or direction of the currents passing through and from different parts of such instruments. It is probably with the view of making this subject more clear, that the electrometer and galvanic multiplier meet with especial attention.

We notice some instances of hasty writing, which are, however, mostly referred to in the errata in the front of the volume. These do not detract from the general value of the work, which we heartily recommend to the attention of medical men, and all others who are in any way interested in pharmaceutical and chemical pursuits. Teachers of chemistry often fail in illustrating their lectures, through want of manipulative tact and experience; and we believe that a perusal of the volume before us, would tend to make the public exhibitions of such persons much more successful than they frequently are. A careful study of it by our druggists, would enable some of them to follow the directions of our dispensatory with more exactness and fidelity than they are in the habit of practising.

---

*Clinical Midwifery. Comprising the histories of five hundred and forty-five cases of difficult, preternatural, and complicated labour. With Commentaries.* By Robert Lee, M. D., F.R.S., Fellow of the Royal College of Physicians, London, etc. etc. First American from the second London edition. 12mo. pp. 238. Lea & Blanchard: Philadelphia, 1849.

To those who have become well grounded in the principles of obstetrics, from a thorough study of the best systematic treatises, clinical reports are of great value. To the inexperienced, they supply, in some degree, data for testing the applicability of the rules they have been taught; and to those who have seen much practice, they afford means for comparison and illustration of their own observations. Still, the circumstances set forth in such reports, are so frequently warped and coloured by the views and foregone conclusions of those from whom they proceed, that in all instances, where they contradict the general testimony of the profession, great caution is necessary in receiving them as elements in our reasoning. We are not about to express any doubts of the truthfulness of the reports before us. With no great reverence for the authority of the author, it must be admitted that he enjoys unusual opportunities for observing difficult and obscure cases, and the reports in the present publication bear upon their face the appearance of truthful expression. We have found, in their perusal, much for useful reflexion to the well read and experienced practitioner, and much that is calculated to mislead the inexperienced, who may trust to the practice set forth in the various cases as examples for them to follow.

While, therefore, we regard this work of Dr. Lee as one of great value for reference in obstetrical statistics, we think it little calculated to enlighten the path of the student. It is true, there is much that he may read with profit, but there is not a little that will conflict with the practice inculcated by some of our best teachers, and it requires an intimate knowledge of the subject to enable the reader to determine how far he may accept the one or reject the other.

---

*Notes of Medical Matters and Medical Men in London and Paris.* By DAVID W. YANDELL, M. D. 8vo. pp. 309. Prentice & Weissinger: Louisville, Ky., 1848.

The volume before us consists of a series of letters, written by the author while pursuing his studies in the chief cities of Europe, and considering the age and inexperience of the writer, and the circumstances under which they were penned, do him great credit.

In the preface, the author modestly remarks: "The letters were written to one of the editors, [of the Western Journal of Medicine and Surgery,] and, at first, without any object beyond his personal gratification. They were deemed by him worthy of publication, and forthwith I was enrolled "Foreign Correspondent" of the Journal. In the midst of engrossing studies, which left me but little leisure, I was induced to continue the correspondence, not more by the evidence afforded me that my contributions were well received, than by the assurance of the working editor that they lightened his onerous labours. The correspondence, commenced and continued in this spirit, has, at length, grown into a volume. As the successive numbers were passing through the press, a few extra sheets were obligingly set apart for the author, by the publishers, and they make the volume now presented to the reader."

Of the propriety and justice of describing the personal appearance, peculiarities and opinions, of living men, without their knowledge and consent, we have never been satisfied. Very often, the reporter has enjoyed but limited opportunities for judging of what he relates, and is, therefore, liable to commit gross, although it may be, unintentional errors; nor can any thing, in our opinion, justify the breach of confidence which is committed by those who disregard the sanctity of the fireside, and expose scenes which their eyes have been permitted to behold only from the promptings of a generous hospitality.

In allusions of this kind, our young author has manifested a delicacy which contrasts favourably with the observations of some older tourists, who have preceded him in the same line of narration; generally, indeed, he has confined himself to a notice of professional rather than personal matters, and when speaking of the eminent men whom he saw, their opinions and practice on matters of professional interest alone are mentioned, and mostly, we think, in a way not calculated to offend.

---

*The Encyclopedia of Chemistry, Theoretical and Practical, presenting a complete and extended view of the present state of Chemical Science.* By JAMES C. BOOTH, Member of the American Philosophical Society, Professor of Technical Chemistry in the Franklin Institute, &c. &c. Assisted by Campbell Morfit, Practical and Analytic Chemist; author of "Applied Chemistry," &c. With numerous engravings. Philadelphia: Carey & Hart.

Number 14 of this Encyclopedia of Chemistry, is the only one which we have seen; but judging from the well known ability of the authors, and from an examination of some of the contents of this number, we are warranted in regarding it as a complete embodiment of the chemical science of the present day, and as such we would recommend it to all those desirous of possessing a book of reference in matters pertaining to chemisty. It is well printed and handsomely illustrated.

*Introductory Lecture before the Anatomical Class of the University of Pennsylvania.* By William E. Horner, M. D., Professor of Anatomy. Delivered October 17th, 1848. Published by the class.

We welcome Professor Horner's introductory with peculiar pleasure. Apart from the claim upon our special notice due to the nature of its theme, it possesses a double interest on this occasion, because it affords to a widely extended circle of friends and pupils, the most gratifying evidence of the improved health and unabated energies of its distinguished author.

Just returning from a summer tour in Europe, which it has been his "fortune to revisit after a lapse of twenty-seven years," and "with a mind occupied with such recent impressions" of stirring incidents, and scenes, the like of which it can be the lot of few lecturers of Dr. Horner's position and engagements to encounter in so short a space of time, he has very naturally favoured his new class with "some of his recollections" as a fitting prelude to his winter demonstrations. We venture to assert that he could offer none more acceptable to the listeners for whom it was intended, or more agreeably instructive to the readers who have to thank those listeners for bringing it within their reach.

A vacation trip to London and Paris, not to mention Edinburgh and Dublin, with perhaps a glance at Belgium and the Rhine, is now-a-days an enterprise of such continued recurrence, that we have ceased to wonder at the ease with which it may be accomplished. But a visit beyond the usual routine, to the more distant high-ways of science in the heart of German Fatherland—a *bona fide* view, however hasty, of the great schools of Vienna, Munich and Berlin, is still something worthy of admiration, even in these days of lightning telegraphs and ocean steamers.

"Three great empires," says Dr. Horner, "present us with the most striking examples of the state of Medical Science in that quarter of the world." And these "three great empires," our honoured friend, with a zeal that smacks little of his years and recent feeble health, has had the courage to invade and explore for the purpose of a candid survey in his capacity of medical professor and practitioner.

Verily, the chance of sharing in a single tithe of the retrospect of such an exploration, would rouse in all their force much feebler yearnings after novelty in introductories than ours. And it would be a labour of love, were the space allowed us in these pages, to trace the traveller, step by step, throughout his deeply interesting and eventful progress. We would gladly follow him from the first start in the thronging world of London, where, true to his vocation, he offers up his earliest tribute of devotion to the memory of Hunter; through the Bavarian Walhalla; the bloody scenes of suffering and slaughter, in the baricaded streets and crowded hospitals of Paris; by the picturesque and legendary beauties of the Rhine and the Danube; by the national glories of the cathedral of Cologne; the splendid palaces of modern letters, art, science and philanthropy at Munich, Vienna and Berlin. No such pleasure is in store for us, however, within our present limits, and we must content ourselves with an earnest recommendation to the present lecture, while we take leave of its author with his own account of the general conclusions to which he has arrived. His idea of the state of medical instruction in the old world is, that though neither the British Union, France, nor Germany, "realize all that we could hope for, yet each has its merits, and we may perhaps say, its superiority, the one over the other, which justly claims our admiration." We learn further, that "there are certain points deserving of our imitation, in the higher attention paid to them, and which attention is permanently secured in the organization and policy of the schools." "Among these," continues Dr. Horner," are organic anatomy, the use of the microscope in normal and pathological investigations, practical chemistry, and, above all others, the nature, observation and treatment of disease in the living body, before a student is permitted to practice medicine in society and graduate fully." "This ascendency of clinical study," he informs us, "is remarkably conspicuous in the German schools." France comes next to Germany in regard to clinical instruction; while in "England" the system is but little better than our own. Germany, its elevated character, its superior, but still unappreciated resources and attraction for the student, have been evidently uppermost in the mind of the Professor from the outset of his lecture. They occupy more than half of the whole address; and, indeed, we are assured at the conclusion, that the author "viewed, with the strongest interest, the several features—social, literary, and scientific—of this great country." "In reflecting on my visit," continues he, "there is but the regret, that I had to do in a few weeks what ought to have taken several months, and might have employed, pleasantly and profitably to the understanding, several years." "As these protracted gratifications were forbidden to myself by my time of life, and the duties of my position, I have thought that in stimulating the curiosity of such of you as propose to complete your education by foreign travel and by foreign study, my narrative would at least be productive of some good; and that it may, in its place, serve to sustain the spirit of improvement in the standard of medical education in our own country, which has been so strongly urged in the proceedings of our three National Medical Conventions." These are sentiments in which every reader of Prof. Horner's excellent discourse must cordially concur, and to which we are happy to render a sincere amen.

---

*Introductory Lecture to the Course of Clinical Instruction in Surgery, at the Pennsylvania Hospital. Delivered November* 1*st,* 1848. By George W. Norris, M. D., one of the Surgeons of that Institution.

This is an exceedingly interesting lecture, and one which we would like to see in the hands of every medical student of this country. It is written in a style, which, if not as elegant or eloquent as that of some of our lecturers, is, at least, remarkable for its good sense and perspicuity; characteristics of good style, but little attended to by some of our most popular lecturers. Throughout this lecture, there is so much genuine modesty of bearing; so much of the true spirit of the philosophical surgeon, that one cannot fail to be impressed favourably with the character of its author. A portion of the lecture is devoted to a brief history of the Pennsylvania Hospital, and to the advantages which this Institution offers for instruction in clinical surgery. In stating to the class, that the managers of the Hospital are not backward in affording every opportunity for medical instruction, consistent with the feelings "of the numerous dependants on their bounty," the lecturer makes the following appropriate request, which should be urged upon a medical class, whenever and wherever in attendance upon clinical instruction.

"So far as you are concerned, I am sure, this proper spirit will be observed, and I will here take the liberty of asking from you, gentlemen, that all marks of applause or of disapprobation may in this theatre be avoided. When you consider that you are here at all times surrounded by the suffering—that there are always in the apartments immediately adjoining us and within the sound of your voices some who might be disturbed by noise, and that the ignorance of others who are not suffering might induce a fear that they are to be brought before you to exhibit their infirmities merely as for a theatrical exhibition, I feel confident that this request need but be mentioned by me in order to secure your observance of it. Our profession is eminently a humane one, and humanity to the poor and friendless, who are here thrown upon us for support and assistance, is shown perhaps more strongly by some sympathy for their sufferings, and the avoidance of everything which may induce them to believe that those sufferings or their feelings are sported with, than in any other manner."

The lecturer earnestly solicits the attention of the students to the minor duties of the surgeon; to the treatment of fractures and dislocations, and to the medical treatment of surgical diseases; and while on the one hand he properly appreciates the value of operative surgery, considering it the last resort of the true surgeon; on the other, he believes "a great and lasting surgical reputation is attained, not by operations, but by a thorough and practical acquaintance with the *principles of surgery.*" In stating that operative surgery has very much diminished with the advance of the science, he says:

"The cultivation of morbid anatomy and pathology, which has within the period mentioned been followed with an ardor previously unknown, by indicating better methods of treatment, as well as by exposing the constitutional origin of what were before regarded as purely local diseases, and by pointing out the grave affections of the general system which so frequently supervene on diseases which in their origin are purely local, has greatly diminished the number of really necessary operations, and I think it would be to the benefit of the student, as well as more honourable to our profession, if instead of dwelling so much upon operative procedures, teachers would more constantly direct the attention of their pupils to the attainment of this really useful knowledge. Ulcers and diseased joints, classes of diseases in which amputation was formerly so common, are now by improved methods of treatment in the great majority of cases cured without resort to operative means. Castration, which in former days was so common an operation in chronic diseases of the testis, is now rarely done—so rarely, that in this hospital, with which I have been connected for fifteen years, and in which many such affections are treated, I have never seen it once performed here. The application of the trephine, too, at one period so frequent, is now rarely resorted to; and within a very short time, the treatment of aneurisms by pressure has been so improved upon by an accurate study of the process employed by nature in her spontaneous cures of that disease, and such an adaptation of the treatment as to imitate her, that there is good reason to hope that in the extremities at least, another bloody operation will be henceforth in many cases deemed unnecessary."

Our knowledge of Dr. Norris' character, convinces us of his sincerity in the inculcation of these judicious views, and we feel satisfied that he will discharge the duties of Clinical Instructor in a manner honourable to himself, and advantageous to the students. Indeed, we consider it a subject for congratulation, that the duties of Lecturer on Surgery, formerly so ably discharged by the lamented Randolph, have devolved upon one so capable as Dr. Norris.

---

*Introductory Lecture to the class of Midwifery and Diseases of Women and Children, in Jefferson Medical College, October 18th,* 1848. By Charles D. Meigs, M. D. *Published by the Class.*

In this lecture which is written in a quaint style, the lecturer draws the distinction between the medical class and the scholar class, and earnestly invites the members of his class not to stop short in their studies, till they reach " the last rank a man can attain, which is that of the scholar." " The physician should be not a gentleman only, but a gentleman and a scholar; for," says he, "it is the scholar who has led his fellow man out of the grossness and ignorance and depravity of his natural state—that has taught him arts and letters, and converted him from being a savage into the more elevated condition of the Christian and gentleman. It is the scholar that has broken asunder the manacles of the woman, and freed her and disenthralled her from being the bond-slave of the man, his beast of burthen, and the mother of his child, to take her station as his co-equal companion. The scholar is man's teacher and guide. He it is who struck the scales from his eyes, and opened his deaf ears, and unsealed his closed senses, and called upon him with his voice, and showed him by his example, the way in which he should walk; invited him to spurn the degradation and bondage of ignorance and depravity, and to come up and be partaker with him of knowledge, which is freedom, and of virtue, which is happiness."

These views are enforced throughout, with that enthusiasm and high toned feeling which characterise the author of this lecture, and we only regret that he has not preferred a plain and easily comprehended style, to the rambling and disconnected one, which he seems purposely to have adopted.

---

*Introductory Lecture to the Course of Chemistry, delivered in Jefferson Medical College, October 18th,* 1848. By Franklin Bache, M. D. Published by the Class.

This lecture comprises a history of chemistry from the first dawning of the science to the present day. The author passes in review the various contributors to chemical lore, beginning with the alchemical writers of the seventh century, and ending with Liebig. With the name of each is connected a brief account of his more important discoveries and theories. It contains an amount of information highly to be coveted by the accomplished physician and chemist, and on this account, as well as for its intrinsic merits as a literary production, we cordially recommend it to the careful perusal of our readers.

